# Supplementary figures and images for: Alanine tRNAs Translate Environment Into Behavior in Caenorhabditis elegans
Source: Front Cell Dev Biol. 2020 Oct 30;8:571359. doi: 10.3389/fcell.2020.571359 (PMC7662486; doi:10.3389/fcell.2020.571359)

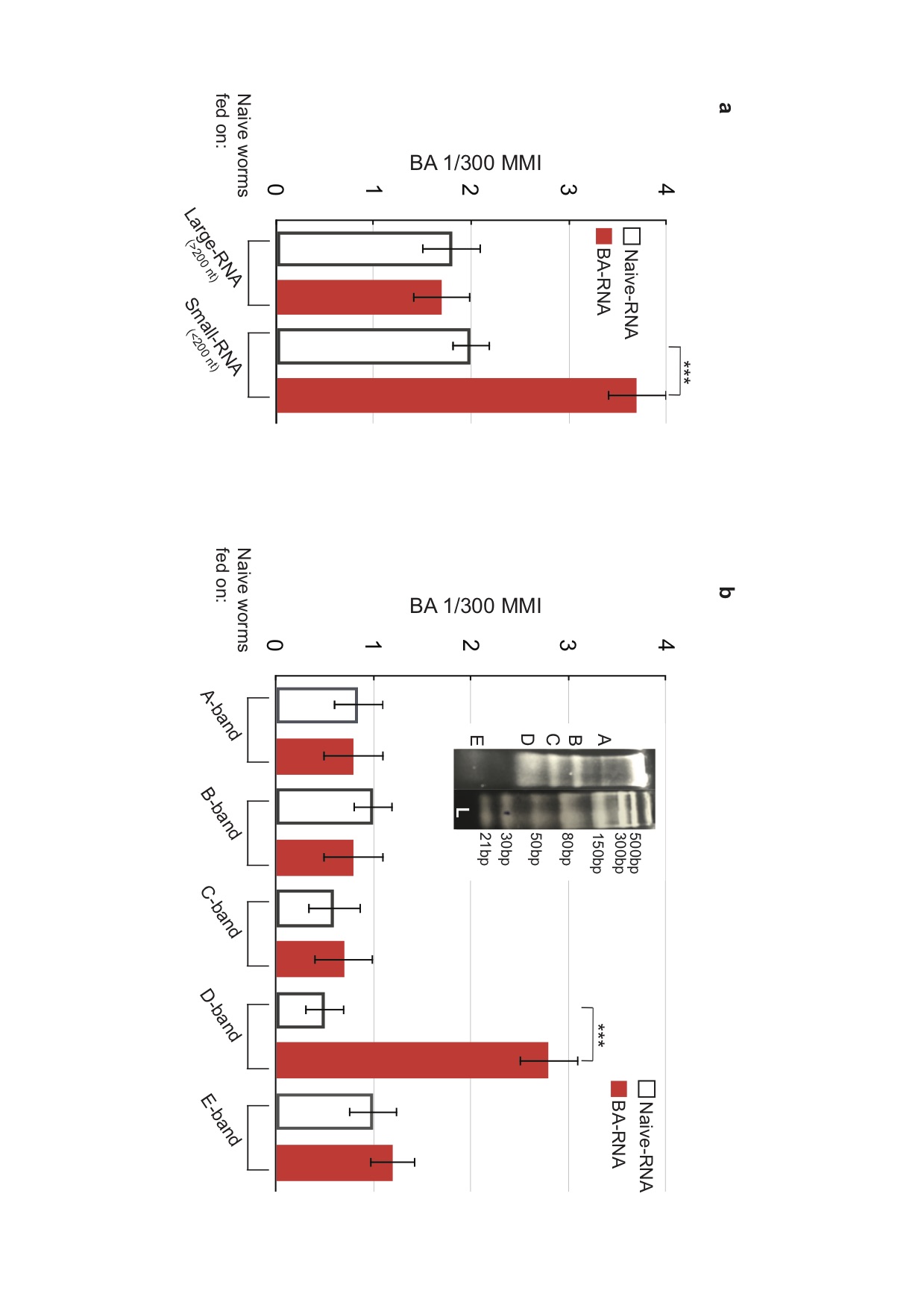

Supplement: Supplementary Figure 1 — A specific population of small RNA transfers olfactory imprinting. (a) RNA from BA-exposed and unexposed naive was fractionated into two populations: the larger than 200 nucleotides fraction (Large-RNA) and the smaller than 200 nucleotides fraction (Small-RNA). Worms fed on 10 ng of Large BA-RNA migrate in a BA gradient as worms fed on 10 ng of Large Naive-RNA. By contrast, worms fed on 10 ng of Small BA-RNA display enhanced BA responses, compared to worms fed on 10 ng of Small Naive-RNA or to worms fed on 10 ng of Large BA-RNA (experimental repeats > 4, ∗∗∗p-value < 0.001). (b) 3.5% low-melting agarose gels fractionate BA-RNA and Naive-RNA into five A to E discrete bands (left side of insert). Naive worms were fed on each of the five gel-purified NA-RNA and BA-RNA populations. Compared BA 1/300 MMI shows that only the BA-RNA band D is able to transfer a BA-imprint to naive (experimental repeats > 4, ∗∗∗p-value < 0.001). (L), NEB double stranded RNA ladder. [file Image_1.JPEG]

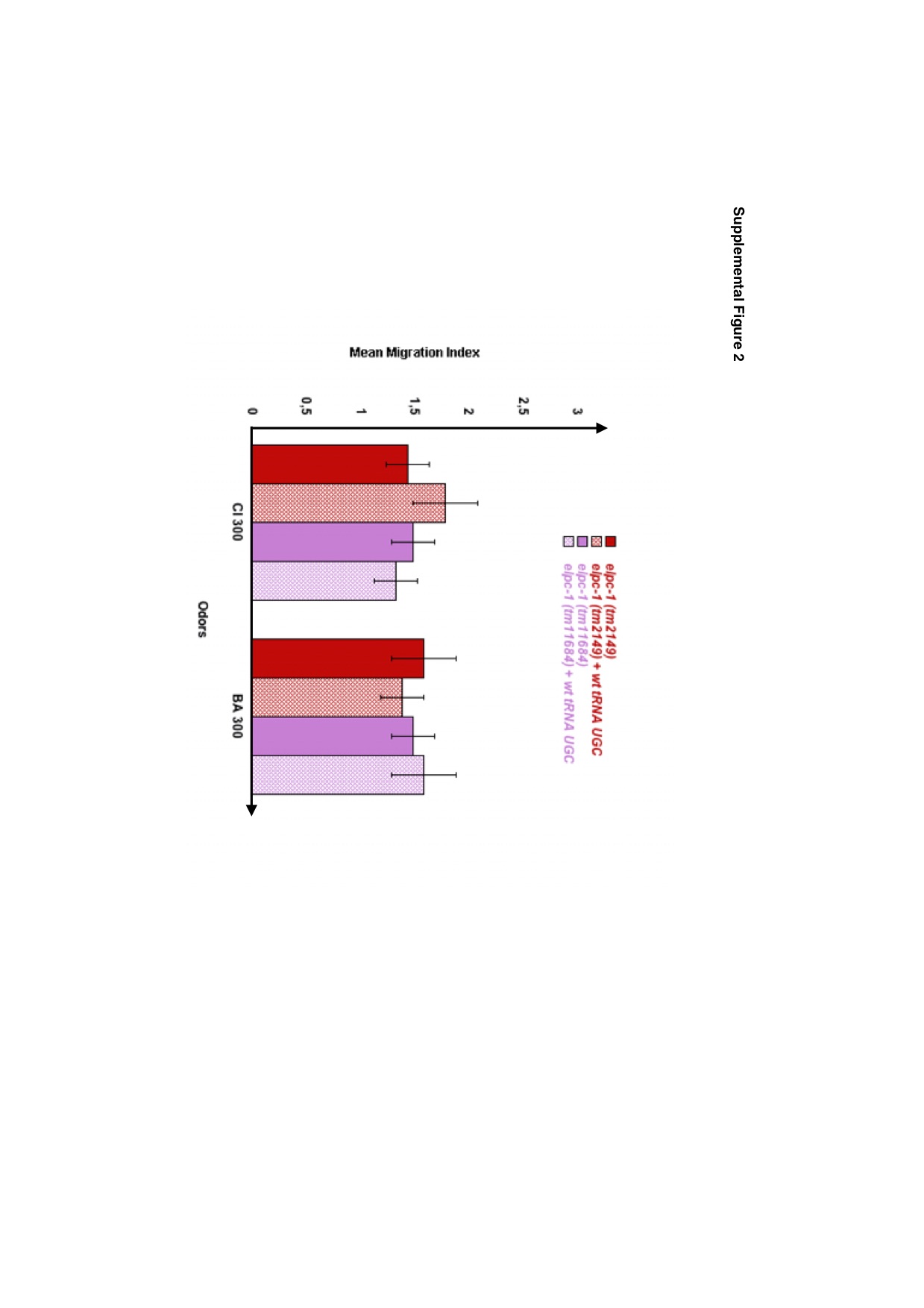

Supplement: Supplementary Figure 2 — Wild-type tRNAAlaUGC has no effect on elpc-1 mutant worms chemo-attraction. Compared chemo-attractive responses of naive or of wild-type tRNAAlaUGC fed elpc-1 (tm2149) and elpc-1 (tm11684) mutant worms. Mean Migration Indices (MMI) in gradients of Citronellol (CI), Benzaldehyde (BA) at the indicated dilutions, were determined as described (experimental repeats ≥ 4, ∗∗p-value < 0.01). [file Image_2.JPEG]

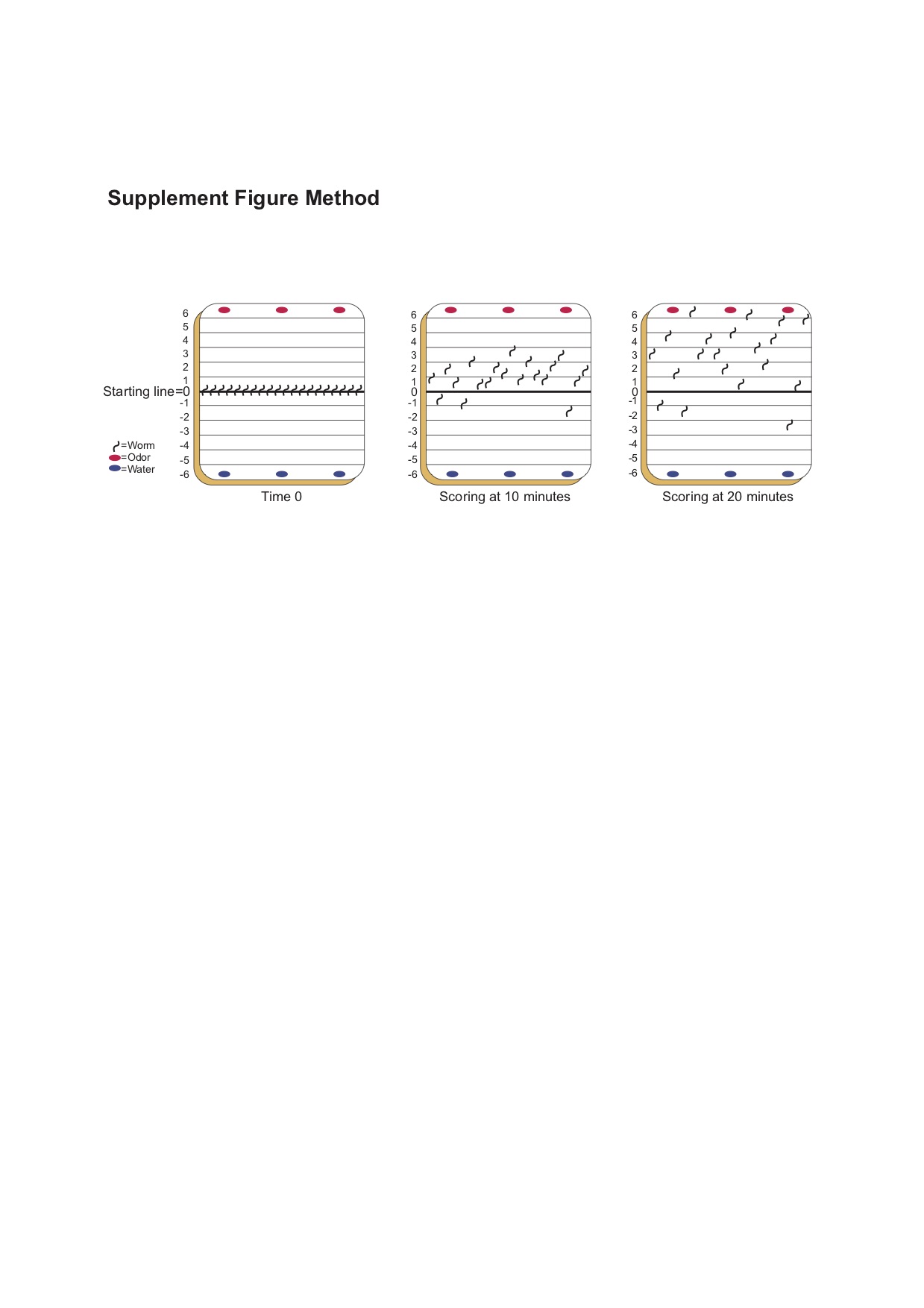

Supplement: Supplementary Figure Method 1 — Schematic outline of the chemotaxis assay. Chemotaxis assays were performed on square plates in order to index the worms’ positions between the starting line (time 0, position 0 cm) and the odor source (position + 6 cm) or water source (position −6 cm). 3 ml of odor solution or water are added to the lids of the plates. 20 synchronized worms are placed at the starting line at time 0. Worms start their migration and their position is carefully noted, usually at four time-points, at 10, 20, 30 and 40 min past time 0. Worms that migrate up the odor gradients are positively indexed (from 0 to 6). Worms that migrate down the odor gradients toward water are negatively indexed (from 0 to – 6). The mean value of all indexed positions at all noted times (in cm from the starting line) of each of the 20 worms involved in the assay is the Mean Migration Index (MMI). [file Image_3.JPEG]
